# Metabolic Products of Linalool and Modulation of GABA_A_ Receptors

**DOI:** 10.3389/fchem.2017.00046

**Published:** 2017-06-21

**Authors:** Sinem Milanos, Shaimaa A. Elsharif, Dieter Janzen, Andrea Buettner, Carmen Villmann

**Affiliations:** ^1^Institute of Clinical Neurobiology, Julius-Maximilians-University of WürzburgWürzburg, Germany; ^2^Department of Chemistry and Pharmacy, Food Chemistry, Emil-Fischer-Center, Friedrich-Alexander-University Erlangen-NürnbergErlangen, Germany; ^3^Department of Sensory Analytics, Fraunhofer Institute for Process Engineering and PackagingFreising, Germany

**Keywords:** Cys-loop receptor, GABA_A_ receptor, linalool, linalyl acetate, oxygenation, patch-clamp

## Abstract

Terpenoids are major subcomponents in aroma substances which harbor sedative physiological potential. We have demonstrated that various monoterpenoids such as the acyclic linalool enhance GABAergic currents in an allosteric manner *in vitro* upon overexpression of inhibitory α1β2 GABA_A_ receptors in various expression systems. However, in plants or humans, i.e., following intake via inhalation or ingestion, linalool undergoes metabolic modifications including oxygenation and acetylation, which may affect the modulatory efficacy of the generated linalool derivatives. Here, we analyzed the modulatory potential of linalool derivatives at α1β2γ2 GABA_A_ receptors upon transient overexpression. Following receptor expression control, electrophysiological recordings in a whole cell configuration were used to determine the chloride influx upon co-application of GABA EC_10−30_ together with the modulatory substance. Our results show that only oxygenated linalool metabolites at carbon 8 positively affect GABAergic currents whereas derivatives hydroxylated or carboxylated at carbon 8 were rather ineffective. Acetylated linalool derivatives resulted in non-significant changes of GABAergic currents. We can conclude that metabolism of linalool reduces its positive allosteric potential at GABA_A_ receptors compared to the significant potentiation effects of the parent molecule linalool itself.

## Introduction

Essential oils form a class of concentrated volatile compounds generated as secondary metabolites in aromatic plants and are characterized by their strong and pleasant odor (Silva et al., 2009; Rowan, 2011). These odorous volatile substances have been widely used in folk medicine and aromatherapy (de Almeida et al., 2004).

Linalool, an acyclic monoterpene, is an important odorous constituent in a series of plant aromas. Amongst others, it is the major component of essential oils (35–51%) obtained from various types of lavender extracts and is widely used for production of fragrances, shampoos, soaps, and detergents (Mitic-Culafic et al., 2009; Carrasco et al., 2016). In addition to the high importance of linalool in perfume industry, several studies on lavender oils have demonstrated the ability of these aromas to improve sleep in elderly people and infants (Hudson, 1996; Field et al., 2008). In line with these observations on humans, a physiological effect on sedation and anxiety-related behavior has been shown in several animal studies following inhalation of linalool with comprehensive analysis of mice in relation to anxiety-related behavior, social interactions, and aggression behavior (Buchbauer et al., 1993; Bradley et al., 2007; Linck et al., 2010; Souto-Maior et al., 2011). The molecular mechanisms behind these actions of linalool as an anxiolytic agent however, remain unsolved. Studies focusing on ion channels were carried out to understand the physiological action of linalool. Using electrophysiological measurements, a non-selective suppression of voltage-gated sodium channels has been demonstrated in olfactory receptor neurons and Purkinje cells (Narusuye et al., 2005). Furthermore, linalool suppressed the function of excitatory glutamate receptors (Silva Brum et al., 2001; Ohkuma et al., 2002). In contrast to suppressive effects, a potentiation of inhibitory GABA_A_ receptors has been previously reported (Kessler et al., 2012, 2014). The observed potentiation at inhibitory GABA_A_ receptors is in agreement with the linalool effect on sedation and anxiety-related behaviors in mice.

In nature or after uptake by ingestion or inhalation, lavender oil undergoes numerous chemical modifications, possibly because lavender oil lacks a natural protection against autoxidation (Hagvall et al., 2008; Christensson et al., 2016). After administration of lavender oil, the main constituent linalool is exposed to enzymatic activity of cytochrome P-450 (CYP76C1) in lung and liver (Chadha and Madyastha, 1984; Boachon et al., 2015). Linalool derivatives such as 8-oxolinalool, 8-hydroxylinalool, and 8-carboxylinalool are formed (Aprotosoaie et al., 2014). Recently, it has been shown that plant leaves also contain cytochromes in order to generate linalool metabolites themselves (Boachon et al., 2015).

Linalool also undergoes acetylation processes. Linalyl acetate metabolism was studied before in *Pseudomonas incognita* (Renganathan and Madyastha, 1983). In addition to acetylation, the C8-moiety of linalyl acetate is susceptible to further oxidation processes giving rise to 8-hydroxylinalyl acetate, 8-oxolinalyl acetate, and 8-carboxylinalyl acetate (Renganathan and Madyastha, 1983). A recent study on the smell characteristics of such structural derivatives of linalool suggested that oxygenation at C8 has a substantial impact on odor properties with 8-oxolinalyl acetate harvesting similar odor properties compared to linalool (Elsharif et al., 2015). The further physiological potential of these numerous linalool derivatives, besides smell, has not yet been investigated with the suggested target molecules. Here, we set out an experimental series to close this gap with the analysis of the modulatory activity of linalool derivatives at GABA_A_ receptors, which are involved in sedative processes in the central nervous system.

GABA_A_Rs belong to the superfamily of Cys-loop receptors and are ligand gated ion channels (Grenningloh et al., 1987). These receptors are mainly present in the mammalian central nervous system (CNS) and are expressed throughout almost the entire brain. Their function in the adult organism is to inhibit incoming action potentials by enhancing the anion concentration of the neuronal cytosol, leading to hyperpolarization of the cell. Binding of two GABA (4-aminobutanoic acid) neurotransmitter molecules to the extracellular domain of the receptor complex are sufficient to induce conformational changes that allow channel opening and a chloride ion influx (Sieghart, 2006). The structure of GABA_A_ channels is determined by five subunits that arrange in a rosette-like structure to form the ion channel pore (Miller and Aricescu, 2014). There are 19 genes that encode for GABA_A_R subunits (α1–6, β1–4, γ1–3, δ, ε, θ, ρ1–2, π) that form GABA_A_ receptor channels (Sieghart, 2006).

Previously, it was demonstrated that processes such as sedation and anxiety are mediated by different GABA_A_R configurations (Low et al., 2000). Thus, GABA_A_Rs represent excellent drug targets for anticonvulsant and sedative agents (Middendorp et al., 2014; Mendonca Junior et al., 2015).

In addition to anticonvulsants, GABA_A_ receptors are allosterically modulated by monoterpenes. Among monoterpenes, bicyclic monoterpenes, and/or carriers of hydroxyl groups harbor the highest positively modulating potential (van Brederode et al., 2016). In a previous study, we have shown that linalool and the bicyclic monoterpenes myrtenol and verbenol identified in *Sideritis* extracts enhanced GABAergic currents 2- to 7-fold (Kessler et al., 2012, 2014). Whereas, linalool is well-analyzed in terms of GABA_A_ modulatory capacity, linalool metabolites or degradation products have never been investigated in the context of GABA_A_ receptor modulation.

Here, we investigate linalool derivatives and their potential action on GABA_A_Rs by overexpressing the ion channel in HEK293 cells. HEK293 cells are widely used for electrophysiological measurements. They are easy to handle and to transfect with plasmid DNA of interest. Receptor overexpression is enabled by transfection of these cells with various GABA_A_ receptor subunits and represents a rather consistent readout to study the neurotropic action of linalool derivatives.

GABA_A_R functionality was investigated upon application of the agonist GABA in the presence and absence of linalool derivatives. The readouts were electrophysiological measurements (patch-clamp) to determine the allosteric potential of the derivatives in terms of modulation of ion channel functionality.

## Materials and methods

### Chemicals

Linalool derivatives were synthesized by Shaimaa Elsharif. All compounds except **L5** have been characterized as described (Elsharif et al., 2015). GABA and linalool were purchased from Sigma-Aldrich (Taufkirchen, Germany). 1,2-Dihydrolinalool (TCI Europe Research Chemicals, Eschborn, Germany), selenium dioxide, ethanol, dioxane and petroleum ether (Sigma-Aldrich, Taufkirchen, Germany), and diethyl ether (Scientific, Loughborough, UK) were purchased for the synthesis of 8-oxo-1,2-dihydrolinalool **(L5)**.

### Synthesis of 8-Oxo-1,2-dihydrolinalool

**Figure 1:**
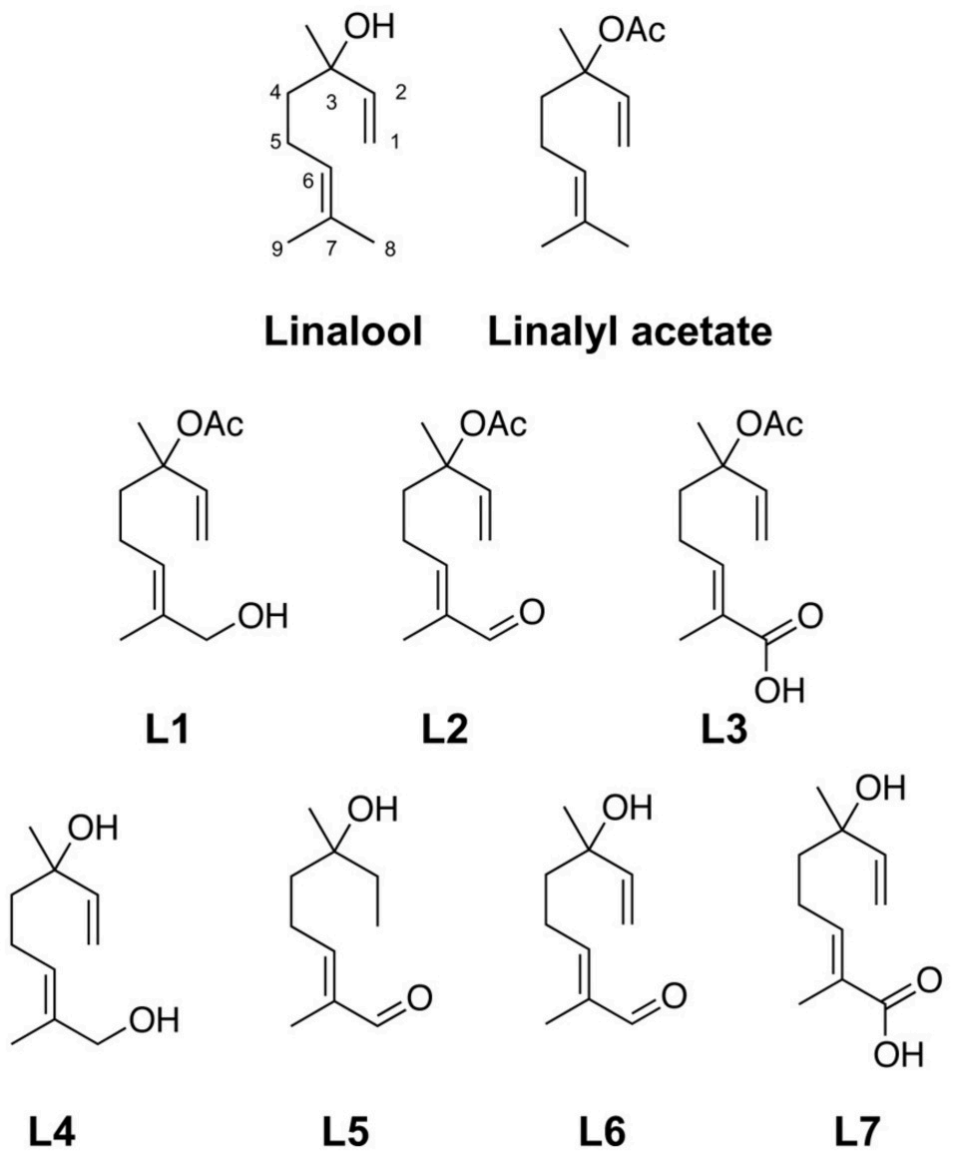
Linalool and linalool metabolites. Linalool a monoterpene alcohol (3,7-dimethyl-1,6-octadiene-3-ol), linalyl acetate and its derivatives. Compounds **L1-3** are acetylated at carbon 3. Compound **L1** (8-hydroxylinalyl acetate), compound **L2** (8-oxolinalyl acetate), compound **L3** (8-carboxylinalyl acetate). Compounds **L4-7** are linalool metabolites substituted at carbon atom 8. Compound **L4** (8-hydroxylinalool), compound **L5** (8-oxo-dihydrolinalool), compound **L6** (8-oxolinalool), compound **L7** (8-carboxylinalool).

Compound 8-oxo-1,2-dihydrolinalool (**L5**) (Figure 1) was prepared following the method of Wakayama et al. (1973). 1,2-Dihydrolinalool (4.9 g, 31.4 mmol) and selenium dioxide (SeO_2_; 3.5 g, 31.4 mmol) were dissolved in 25 mL of dioxane/ethanol 9:1 (v/v) and the solution was heated at 80°C for 5 h. After removal of selenium deposit by double filtration, the solvent was removed under reduced pressure using a rotary evaporator. The residue was purified twice by flash chromatography on silica gel 60 (Merck, Darmstadt, Germany) with petroleum ether/diethyl ether 1:4 (v/v) to afford 1.182 g (22.2 %) of **L5** as a reddish-orange oil:

^1^H NMR (600 MHz, CDCl3-d) δ 0.92 (t, J = 1.00 Hz, 3 H) 1.19 (s, 3 H) 1.31–1.43 (m, 2 H) 1.53 (q, J = 7.30 Hz, 2 H) 1.59–1.64 (m, 2 H) 1.75 (s, 3 H) 6.52 (t, J = 1.00 Hz, 1 H) 9.38 (s, 1 H); ^13^C NMR (151 MHz, CDCl3) δ 195.26, 154.94, 139.20, 72.54, 39.48, 34.49, 26.18, 23.68, 9.11, 8.22; MS (EI) *m/z* (%) (rel int) 170 [M^+^] (1), 155 (3), 141 (6), 123 (10), 95 (82), 83 (41), 73 (94), 67 (44), 55 (46), 43 (100).

### Cell lines

The HEK293 cell line (Human embryonic kidney cells) was purchased from ATCC (Wesel, Germany) and grown in Earle's minimal essential medium (MEM) supplemented with 10% fetal calf serum, 200 mM GlutaMAX, 100 mM sodium pyruvate and 50 U/mL penicillin/streptomycin (Sigma-Aldrich, Taufkirchen, Germany) under standard growth conditions at 37°C and 5% CO_2_.

### Transfection of cell line

HEK293 cells were transiently transfected using a modified calcium-phosphate precipitation method. Plasmid DNAs of GABA_A_ α1, β2, γ2 subunits and GFP were mixed in a ratio of 1:1:2:1 and supplemented with 2.5 M CaCl_2_ and 2x HBS buffer (50 mM HEPES, 12 mM glucose, 10 mM KCl, 280 mM NaCl, 1.5 mM Na_2_HPO_4_, pH 6.98) followed by 20 min incubation at room temperature. The transfection solution was applied onto the cells for 6 h. Cell medium was replaced by fresh medium to reduce transfection stress. Electrophysiological recordings were performed 48 h post-transfection. To control for transfection efficiency, a GFP plasmid was cotransfected when cells were used for electrophysiological analysis. Using a fluorescence microscope, green fluorescent cells were detectable. It is expected that cells transfected with GFP also have taken up other plasmid DNAs provided in the same transfection solution. Only green cells were used for electrophysiological analysis.

### Immunocytochemical stainings

Cells were transfected with GABA_A_ α1 and β2 subunits together with pDsRed-Monomer-Mem 1:1:0.5. pDsRed-Monomer-Mem (Clontech, Mountain View, CA, USA) encodes a fusion protein consisting of neuromodulin (GAP-43) and a red fluorescent protein used as plasma membrane marker. To proof surface expression, the α1 subunit was stained following fixation with 4% PFA and three washing steps with PBS (phosphate-buffered saline). A blocking step was included for 1 h using 5% normal goat serum in PBS. Cells were not permeabilized to allow staining of surface membrane proteins only. As primary antibody a rabbit-anti GABA_A_R α1 (ST-J93186-200, Biotrend, Köln, Germany) was used in a 1:500 dilution. After 1 h of incubation, cells were washed three times with PBS before the secondary antibody goat-anti rabbit Alexa488 (Dianova, Hamburg, Germany) was applied for 30 min. After a final wash, a 5 min incubation step with DAPI (1:20,000) was done before cover slips were mounted with Mowiol (Sigma-Aldrich, Taufkirchen, Germany). Immunocytochemical stainings were imaged using the Olympus IX-81 confocal microscope (Olympus, Hamburg, Germany).

### Electrophysiological measurements

Forty-eight hours after transfection, electrophysiological measurements were carried out. The electrophysiological setup is supplied with a fluorescence microscope, which easily allowed detection of GFP positive cells. It is believed that GFP positive cells also have taken up other plasmid DNAs provided by the same transfection solution. All GFP positive cells selected for electrophysiological recordings showed an inward chloride current following application of the agonist GABA demonstrating the successful cotransfection of GABA_A_ receptor subunits forming functional ion channels.

Whole cell recordings from transfected HEK293 cells were performed by application of ligand (GABA) at various concentrations (0.3, 1, 3, 10, 30, 300, 1,000 μM) to estimate the concentration where half-maximal channels responded (EC_50_). Current signals were amplified with an EPC-10 amplifier (HEKA, Lambrecht, Germany). The aroma substances (2 mM) were co-applied for 500 ms together with GABA at EC_10−30_ to the same cell. Cells measured in the same experiment with GABA and GABA + compound were compared and taken for analysis and data presentation. Mean absolute current values of all cells measured are shown in Table 1.

**Table 1:** Electrophysiological properties of modulated GABA_*A*_ receptor of the α1β2γ2 subtype.

**Compound**	**I_abs_ [*n*A] ± SEM**	**I_rel_ [%] ± SEM**	**I_abs_ [*n*A] ± SEM**	**I_rel_ [%] ± SEM**
	10 μM GABA	10 μM GABA	10 μM GABA + compound	10μM GABA + compound
Linalool	0.4 ± 0.07	100 ± 15	0.7 ± 0.1	160 ± 14
Linalyl acetate	0.4 ± 0.07	100 ± 15	0.6 ± 0.3	136 ± 16
L1	1.5 ± 0.8	100 ± 53	1.0 ± 0.4	68 ± 42
L2	1.1 ± 0.4	100 ± 37	1.3 ± 0.4	122 ± 33
L3	0.6 ± 0.1	100 ± 12	0.5 ± 0.1	95 ± 16
L4	0.5 ± 0.1	100 ± 16	0.5 ± 0.1	82 ± 26
L5	0.8 ± 0.4	100 ± 37	1.3 ± 0.5	144 ± 58
L6	0.5 ± 0.2	100 ± 48	0.8 ± 0.3	166 ± 35
L7	1.1 ± 0.5	100 ± 35	1.1 ± 0.3	99 ± 29

Recording pipettes were fabricated from borosilicate capillaries with an open resistance of about 4 MΩ. Cells that were sealed with a resistance of 0.5–1 GΩ and leak currents below –300 pA were taken into the data pool. The capacity of recorded cells was in the range of 10–15 pF. All current responses were measured at a holding potential of –60 mV. The experiments were carried out at room temperature. The extracellular buffer consisted of 137 mM NaCl, 5.4 mM KCl, 1.8 mM CaCl_2_, 1 mM MgCl_2_, 11 mM EGTA, 10 mM HEPES, with a pH adjusted to 7.4 with NaOH. The internal buffer was 120 mM CsCl, 20 mM N(Et)_4_Cl, 1 mM CaCl_2_, 3 mM MgCl_2_, 11 mM EGTA, 10 mM HEPES with a pH adjusted to 7.2 with CsOH.

### Statistical analysis

Concentration-response curves were constructed from the peak current amplitudes obtained with at least seven appropriately spaced concentrations in the range 0.3–1,000 μM GABA. Using a non-linear algorithm (Microcal Origin), concentration-response data were first analyzed using the following Hill equation *I*_*GABA*_*/I*_*sat*_ = [GABA]^*n*^^Hill^/[GABA]^*n*^^Hill^ + ${\text{EC}}_{50}^{\text{nHill}}$ where *I*_*GABA*_ refers to the current amplitude at a given GABA concentration, *I*_*sat*_ is the current amplitude at saturating concentrations of GABA, EC_50_ is the GABA concentration producing half-maximal current responses, and *n*_Hill_ is the Hill coefficient.

The significance levels for recorded absolute currents were calculated using the students *t-*test. Data presented do always show mean values. The error bars refer to SEM (standard error of the mean) values. Significance level are ^*^P < 0.05, ^**^P < 0.01, ^***^P < 0.001.

## Results

GABA_A_ receptors are modulated by terpenoid substances. Linalool, a major component of lavender oil harbors GABA_A_ receptor modulatory potential. Here we investigate derivatives of linalool that can be biosynthesized by plants or formed by biotransformation processes of linalool in the liver or lung following inhalation of linalool or food intake. These substances have already been characterized in the context of their structure odor-activity relationships (Elsharif et al., 2015).

### Generation of linalool derivatives

Linalool is a monoterpene alcohol (3,7-dimethyl-1,6-octadiene-3-ol), while linalyl acetate is substituted with an acetyl moiety at carbon atom 3 (C3) (Figure 1). The conjugated compounds can be divided into two groups. The first group of compounds (**L1-L3**) (Figure 1) contains two modifications within the linalool structure. (i) **L1-L3** are acetylated at carbon atom 3 (C3). (ii) These acetylated compounds carry an additional modification on carbon atom 8 (C8). Both modifications generate compounds **L1** (8-hydroxylinalyl acetate), **L2** (8-oxolinalyl acetate), and **L3** (8-carboxylinalyl acetate) (Figure 1). The second group of linalool derivatives is oxygenated on C8, while the hydroxy-group at C3 remained. This series results in compounds **L4** (8-hydroxylinalool), **L5** (8-oxo-1,2-dihydrolinalool), **L6** (8-oxolinalool), and **L7** (8-carboxylinalool) (Figure 1). **L5** is the only linalool derivative with two substitutions, an oxygenation at C8 and a reduction at C1-C2 generating 8-oxo-1,2-dihydrolinalool.

### GABA_A_ α1β2γ2 receptor complex used for functional analysis is expressed at the cell surface

**Figure 2:**
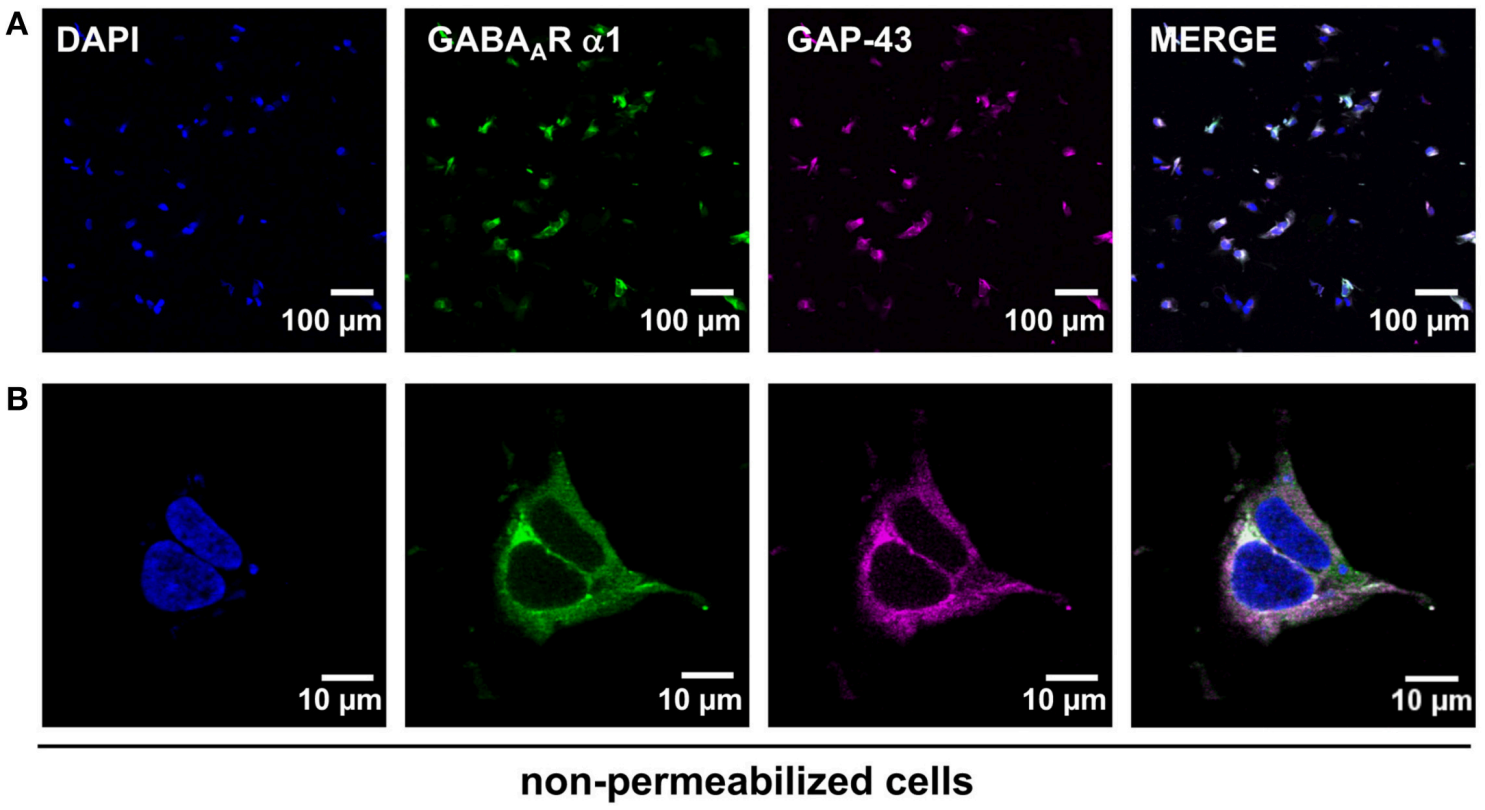
HEK293 transiently expressing GABA_A_ α1β2 receptors and GAP-43 fused with red fluorescent protein. **(A)** Upper lane demonstrates transfection efficiency controlled by co-transfection of the membrane marker protein GAP-43 fused to dsRed (magenta). The α1 subunit of the GABA_A_ receptor was stained specifically at the cellular surface (green). The merged picture represents co-localization resulting in a white signal of both the GABA_A_ α1 receptor subunit and GAP43 (magenta) at the plasma membrane. **(B)** The lower lane shows an enlarged image of a single stained cell. DAPI staining was used to mark the nucleus of the cell (blue), α1 subunit is marked in green and GAP-43 in magenta.

The GABA_A_R complex requires cell surface expression in order to allow functional ion channel analysis. Functional heteromeric GABA_A_ receptor channels are formed by α1β2 and α1β2γ2 subunits (Luger et al., 2015). Therefore, the expression of the α1 subunit was tested by immunocytochemical stainings (Figure 2). The cotransfection of GAP-43 allowed an estimation of the transfection efficiency in addition to its function as cellular membrane marker. The transfection efficiency was determined to around 60% (Figure 2A, lower magnification, right merged image). The GABA_A_ α1 subunit is transported to the cellular surface and co-localized with the membrane marker GAP-43 (Figures 2A,B). The enlarged pictures demonstrate the precise surface expression by the observed ring-like structure at the outer cell surrounding (Figure 2B). This ring-like structure is only observed without permeabilization of the cells during staining procedure. Since homomeric α1 receptors have never been shown to form functional GABA_A_ receptors, the proof of heteromeric receptor functionality can only be given by electrophysiological analysis.

### GABA_A_R modulation by linalool derivatives

**Figure 3:**
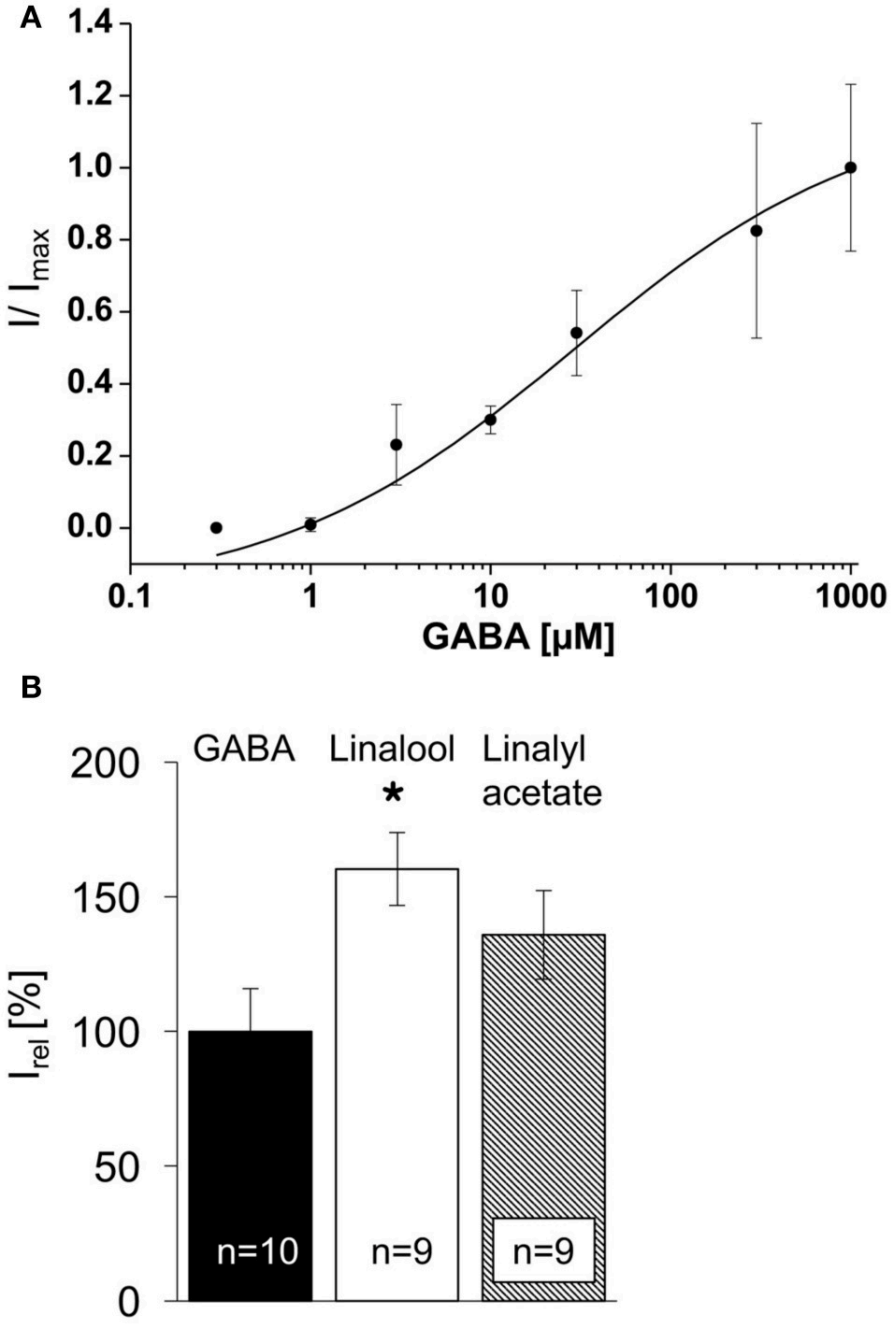
GABA_A_ α1β2γ2 receptor EC_50_ determination for the agonist GABA. **(A)** Calculation of half-maximal receptor activation using seven different concentrations of the agonist (0.3–1,000 μM). The EC_50_ was determined at 37 ± 0.4 μM GABA, while EC_10−30_was calculated to 10 μM. **(B)** The black bar illustrates the mean relative current of GABA_A_ receptors α1β2γ2 at 10 μM GABA. Potentiation of GABAergic currents by linalool (2 mM) at EC_10−30_GABA concentrations (10 μM) refers to the white bar, ^*^P < 0.05; linalyl acetate (striped bar); *n* = number of cells recorded.

All measurements were carried out 48 h post-transfection. To analyze GABA_A_R functionality, a series of GABA concentrations (0.3–1,000 μM) was applied to patched GFP-positive transfected HEK293 cells in an α1β2γ2 configuration to determine the EC_50_ value 37 ± 0.4 μM (Figure 3A). Most published data analyzing ion channel modulators use EC_5−10_ concentrations of agonist to come up with highest effects observed for the modulating agents (Khom et al., 2007). Higher GABA concentrations up to saturating levels will highly reduce the potentiating effects of the compounds. We used an EC_10−30_ GABA concentration (10 μM GABA) for physiological analysis (Figure 3B). The currents observed after 10 μM GABA application were normalized to 100% (Figure 3B, black bar). Linalool (2 mM) or linalyl acetate were co-applied with 10 μM GABA. The observed enhanced relative currents were quantified to 160 ± 14% for linalool (white bar) and 136 ± 16% for linalyl acetate (striped bar; Figure 3B, Table 1). The 1.6-fold increase in I_rel_ following linalool application corresponded to a significant elevation (^*^P < 0.05). The data obtained for linalool are in line with the described potency reported previously in the literature (Kessler et al., 2014).

**Figure 4:**
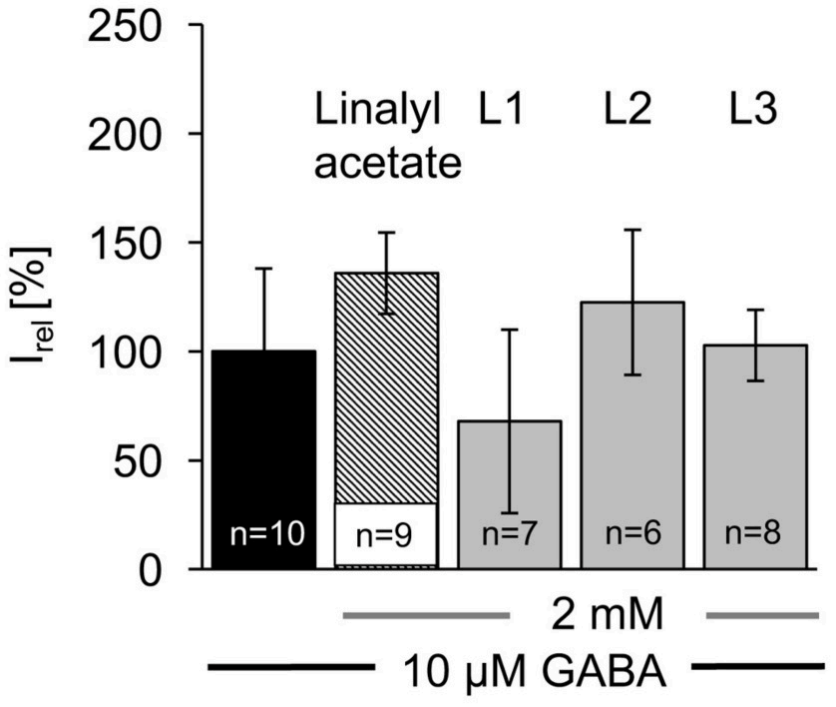
Acetylated linalool derivatives do not potentiate GABAergic currents in HEK293 cells. Ten micromolars of GABA were applied to α1β2γ2 receptors and refers to 100% (100 ± 15% GABA application, black bar; relative to GABA + linalyl acetate). Acetylated linalool compounds such as linalyl acetate (striped bar), **L1 (**8-hydroxylinalyl acetate), **L2** (8-oxolinalyl acetate), **L3** (8-carboxylinalyl acetate) used in a 2 mM concentration were co-applied with GABA (gray bars), *n* = number of cells recorded.

The linalool derivatives (2 mM) were co-applied on the cell after a first application of the agonist alone (10 μM) and resulted in an inward chloride current (Figure 4, Table 1). Application of the acetylated compounds **L1–L3** together with GABA did not result in significant changes of the GABAergic currents (Figure 4). Compound **L1** (8-hydroxylinalyl acetate) resulted in a decreased relative current (I_rel_) of 68 ± 42%, while compound **L2** (8-oxolinalyl acetate) reached 122 ± 33% of a GABA-evoked current. Current values for **L1** demonstrated that among different cells a large variability exists otherwise this derivative would be considered a negative modulator. **L3** (8-carboxylinalyl acetate) was with 95 ± 16% almost indistinguishable from GABA application only (Figure 4, for absolute current values see Table 1).

**Figure 5:**
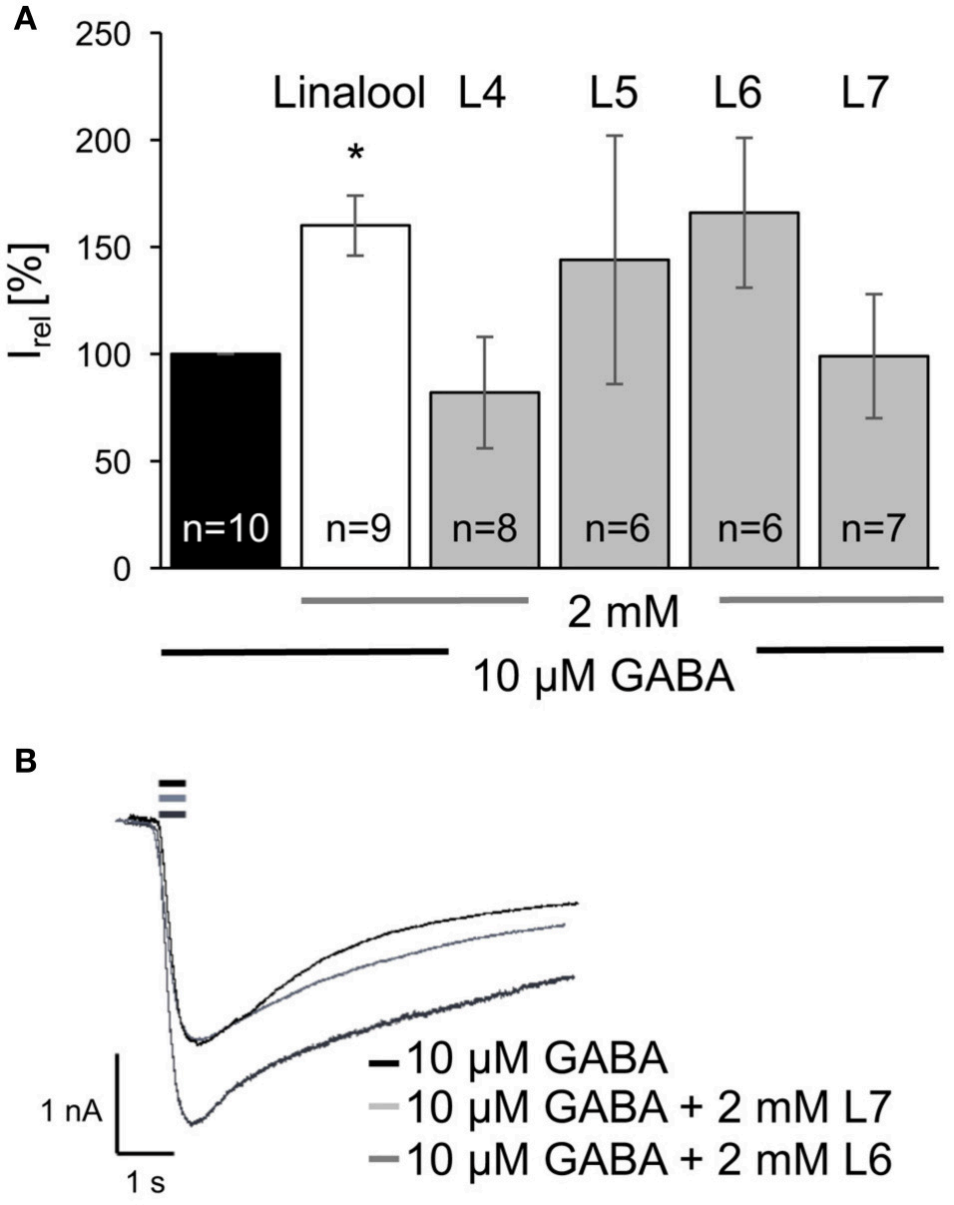
Oxygenated linalool derivatives potentiated GABAergic currents in HEK293 cells. **(A)** 10 μM GABA applications are nominated to 100% (100 ± 15% GABA application relative to GABA + linalool, black bar), linalool (white bar), ^*^P < 0.05. Compounds **L4** (8-hydroxylinalool) and **L7** (8-carboxylinalool) did not enhance or reduce GABA-evoked currents significantly. Compounds **L5** (8-oxo-dihydrolinalool) and **L6** (8-oxolinalool) enhanced GABAergic currents (gray bars). **(B)** Representative current traces detected from transfected HEK293 cells with GABA_A_ receptor subunits α1β2γ2 following an application of 10 μM GABA for 500 ms (black trace) or a co-application on the same cell of 10 μM GABA + **L6** (dark gray trace, potentiating) or **L7** (light gray trace, not potentiating), small bars above the traces refer to the time the agonist ± modulator were applied to the cell, *n* = number of cells recorded.

In case of the oxygenated linalool products differences in modulation were seen. Compound **L4** (8-hydroxylinalool) reached 82 ± 26% of the relative currents observed after GABA application alone. Again, a hydroxy group at C8 rather generated reduced GABAergic currents. In contrast, compounds **L5** (8-oxo-1,2-dihydrolinalool) and **L6** (8-oxolinalool) showed a positive modulatory effect at GABA_A_ receptors of the α1β2γ2 configuration. This receptor configuration refers to 65% of all GABAergic neurons *in vivo*. **L6** application generated GABAergic currents, which were 1.66-fold increased (166 ± 35% compared to GABA alone, Figures 5A,B, Table 1). The modulatory potency of **L6** was comparable to linalool, but did not reach significance. Coapplication of GABA together with **L5** had also a pronounced effect with 144 ± 58% of GABA-induced currents (Figure 5A). Compound **L7** (8-carboxylinalool) had no significant effect with 99 ± 29% of GABA activity when applied (Figures 5A,B).

## Discussion

Linalool is well-known for its odor potential in perfume industry and aromatherapy. Due to the calming effect of linalool, this acyclic monoterpene has been investigated in studies on sedation and anxiety in animal models (Linck et al., 2010; Souto-Maior et al., 2011). It may be tempting to assume that smell potency and pleasantness of a compound might be related to further physiological action, especially balancing and relaxation effects or anxiolytic potential. Well-being as induced by the sense of smell and odorous substances, however, most likely needs to be separated into different modes of action and temporally as well mechanistically independent pathways.

While odorants act in humans first at the periphery with activation of G-protein coupled odorant receptors, i.e., metabotropic receptors, their mode of allosteric modulation at GABA_A_ receptors differs as these ionotropic receptors form structurally completely different physiological entities in higher brain regions. Distinct GABA_A_ receptor configurations in the central nervous system have been shown to mediate processes, such as sedation and anxiolytic effects (Low et al., 2000). A modulation of GABA_A_ receptors of the sedative α1β2γ2 subtype by linalool was proven in *Xenopus* oocytes and an allosteric mechanism for the observed interaction was postulated (Kessler et al., 2014). Here, the same GABA_A_ receptor subtype was used to analyze linalool and its derivatives after acetylation of linalool at C3 and further oxygenation at C8 of linalool and linalyl acetate. Following control of receptor expression, the determination of the EC_50_-value for half-maximal receptor activation was comparable to EC_50_-values described in the literature (Sieghart, 2006). Maximal neurotransmitter application will superpose the modulatory efficacy of terpenes at GABA_A_ receptors (Hossain et al., 2002). A GABA concentration of 10 μM corresponding to EC_10−30_ was used to obtain maximal modulation by the volatile compound used. At the used GABA_A_ receptor configuration α1β2γ2, linalool potentiated the GABAergic responses significantly by 1.7-fold similar to previous observations (Kessler et al., 2014).

Oxidative and polymerization processes of essential oils may result in loss of quality and pharmacological properties (Turek and Stintzing, 2013). The linalool skeleton is characterized by double bonds and the C3 hydroxy group making it vulnerable to further modifications including derivatization (Raguso, 2016). The importance of C3 in linalool as a main contributor to odor quality has recently been shown (Elsharif et al., 2015). Acetylation at C3 resulted in low odor potency. This effect can be compensated by a further modification of linalool at the C8 atom. Oxidation at C8 generating 8-oxolinalyl acetate (**L2**) and 8-carboxylinalyl acetate (**L3**) rescued odor potency while hydroxylation at C8 did not positively contribute to odor potency (Elsharif et al., 2015). The modulation of the GABA_A_ receptor functionality was also affected by modifications of the linalool structure. Although changes in GABAergic currents were small and data did not reach significance, 8-oxolinalyl acetate (**L2**) showed a tendency to positively modulate GABA_A_ receptors with a 1.2-fold increase whereas 8-carboxylinalyl acetate (**L3**) did not modulate the receptor. Hydroxylation at C8 of linalyl acetate led to a reduction of the overall GABAergic responses. Thus, hydroxylation of linalyl acetate does not only give rise to low odor quality but also to lack of modulating potential at GABA_A_ receptors.

Oxidation via cytochrome P-450 of linalool occurs in the liver of animals but similar oxygenation reactions have also been depicted in plants (Chadha and Madyastha, 1984; Boachon et al., 2015). The determined oxygenated derivatives of linalool in animals or plants were 8-oxolinalool (**L6**), 8-hydroxylinalool (**L4**), and 8-carboxylinalool (**L7**) (Chadha and Madyastha, 1984; Aprotosoaie et al., 2014). Moreover, odorants and other volatile substances can also be metabolized right at the periphery within the nasal cavity in the frame of peri-receptor events, thereby potentially generating novel substances and degrading others. This process has been shown to go along with changes in smell character and intensity of specific substances (Schilling, 2017). Terpenoid substances can also undergo heavy biotransformation prior to metabolism following ingestion and passage of the gastro-intestinal tract (Heinlein and Buettner, 2012). In the gut, chemical transformation processes are primarily associated with the acidic conditions in the stomach. A series of other terpenoid derivatives has been determined following these transformation pathways, e.g., linalool degraded to geraniol, nerol, and α-terpineol (Heinlein and Buettner, 2012). Accordingly, biotransformation and bioavailability are important aspects when discussing potential physiological effects of aroma substances.

The odor-function relationship analysis revealed that oxygenation at C8 of linalool and 8-hydroxylinalool showed reduced odor potential. 8-Carboxylinalool was odorless (Elsharif et al., 2015). Yet, it needs to be kept in mind that additional nasal biotransformation processes may occur in smell perception of humans, in some cases. It is not fully clear if these smell effects do only relate to the respective substance or potentially also some derivatives thereof. Nevertheless, it is interesting to note that analysis of the allosteric modulation at the GABA_A_ receptor subtype α1β2γ2 demonstrated that 8-hydroxylinalool (**L4**) and 8-carboxylinalool (**L7**) lost the ability to potentiate GABAergic responses. In contrast, 8-oxolinalool (**L6**, 1.6-fold) and 8-oxo-1,2-dihydrolinalool (**L5**, 1.4-fold) kept at least in part the ability to potentiate GABA_A_ receptors but did not reach significance as observed for linalool. In summary, the hydroxy group at C3 in linalool is obviously important for the positive allosteric modulation at inhibitory GABA_A_ receptors. The structural modification at C8 by oxygenation might be promising to further increase modulatory potency maybe by combining this oxygenation with other structural modifications, e.g., at C1/C2. These data might also have an impact on structural alterations of other monoterpenes or bicyclic terpenes, which have also been pointed out to allosterically interact with GABA_A_ receptors (Granger et al., 2005; Kessler et al., 2014). Our group recently investigated the effect of structural modification on smell parameters of other related terpenoid substances, namely geraniol, nerol, β-citronellol, and their related acetates (Elsharif and Buettner, 2016). Of these, β-citronellol has previously also been shown to exert GABAergic modulatory activity (Kessler et al., 2014). Accordingly, it will be interesting to investigate if their related analogs show comparable effects in GABAergic modulatory capacity. Processing of monoterpenes following ingestion or inhalation accordingly becomes an important issue since these substances also target other ligand-gated ion channels. The allosteric interaction between linalool and GABA_A_ receptors is hypothesized to occur by a direct interaction with the transmembrane domains of the receptor embedded in the hydrophobic lipid bilayer of neuronal cells and thus, accessible by the highly hydrophobic monoterpene (Khom et al., 2007). The analyzed metabolic products of linalool increased solubility of the compounds. We can conclude from our study that increasing the compounds solubility results in a reduction of GABA_A_ receptor modulation. To keep the balance between lipophilicity and solubility is therefore important in novel screens for modulators maintaining their natural odor and physiological potential.

In our previous study we could show that linalool and 8-oxolinalyl acetate (**L2**) were both the most intense odorants of all substances investigated, with odor thresholds as low as 3 and 6 ng/L air, respectively (Elsharif et al., 2015). Interestingly, both compounds also showed comparable GABA_A_ modulatory activity. However, in this context it is important to note that smell perception, i.e., the “peripheral” effect of such substances, can be highly variable between individuals. Amongst others, we could show that such odorants are, at times, perceived with highly variable odor thresholds and, vice versa, odor intensities by different subjects, and can even elicit different smell impressions. It is hypothesized here that, in contrast to this individually variable sensory effect, the pharmacological effect of neurotropic action, as mediated via GABA_A_ receptor expression in transfected cells, rather represents a consistent readout. Moreover, several *in vivo* studies have demonstrated common physiological effects on sedation and anxiety-related behavior in mice following inhalation of linalool (Buchbauer et al., 1993; Bradley et al., 2007; Linck et al., 2010; Souto-Maior et al., 2011). Thus, our study suggests that a combined approach of physiological determination of odor and their metabolite neurotropic activities ideally by high-throughput techniques combined with personalized odor perception profiles represent a future perspective for aromatherapy and anxiety-related diseases. All these considerations, however, require much more detailed combined studies in the future, likewise involving human studies, with specific focus on single individuals.

## Author contributions

Participated in research design: CV, AB. Conducted experiments: SM, DJ. Substances synthesized by SE, Writing paper: CV, SM, AB.

### Conflict of interest statement

The authors declare that the research was conducted in the absence of any commercial or financial relationships that could be construed as a potential conflict of interest.

## Abbreviations

GABA_A_R: GABA_A_ receptor
C3: carbon atom 3
C8: carbon atom 8
GABA: 4-aminobutanoic acid.
